# Comment on: Telemedicine in the management of rheumatoid arthritis: maintaining disease control with less health-care utilization

**DOI:** 10.1093/rap/rkab032

**Published:** 2021-05-06

**Authors:** Diego Benavent, Luis Fernández-Luque, Victoria Navarro-Compán, Alejandro Balsa, Chamaida Plasencia

**Affiliations:** 1 Rheumatology Service, Hospital Universitario La Paz-IdiPaz, Madrid, Spain; 2 Adhera Health Inc., Palo Alto, CA, USA

 

Dear Editor, we read with great interest the manuscript by Müskens *et al*. In a groundbreaking study in rheumatology, they evaluate the implementation of an eHealth platform and a self-management outpatient clinic in patients with RA. Following the use of the platform, the mean number of outpatient clinic visits decreased, and this was accompanied by a significant decrease in DAS28. As they concluded, real-world implementation of remote monitoring can reduce the number of hospital visits, while maintaining disease control. Their publication provides interesting evidence for assessing new evidence-based digital tools in health care [1].

Currently, decisions on therapeutic strategy are based mainly on compound activity indices [2]. However, a patient's perception of their disease activity usually differs from that of their physicians [3]. For instance, it has been shown that health-care professionals may underestimate pain in RA, leading to potential divergences between patients and doctors [4]. An added value of the digital solution used in this study is that it includes patient-reported outcomes measures (PROMs) [1]. Evidence about correlation between PROMs and disease activity scores in RA is increasing [5]. Hence, several projects using PROM-based instruments offer the opportunity for custom-made off-hospital monitoring that gets patients more engaged in the self-management of their disease [6].

Regarding PROMs, one type that might be particularly interesting for assessment of patients is ecological momentary assessment (EMA). EMA is based on repeated sampling of a subject's current behaviour and experience, which enables data collection with high frequency and within the patient's normal environment [7]. This reduces memory biases and increases the reliability of data, especially for measurement of subjective and changing aspects, such pain. EMA has rarely been used in rheumatic and musculoskeletal diseases (RMDs) studies, but it has recently gained widespread interest for chronic diseases and diseases with recurrent flares [8]. Another type of instrument that can be used to place patients at the centre of research and evaluate clinical care is patient-reported experiences measures (PREMs) [9]. PREMs are tools that report patient satisfaction scores with a health service. They are used to monitor patient feedback regarding overall patient experience of health care. PREMs have shown correlations with patient satisfaction and safety and can be used to assess clinical effectiveness and economic efficiencies in health care [10].

The manuscript by Müskens *et al.* [1] highlights that the time to understand what happens to the patient outside of the medical appointment is now. The coronavirus disease 2019 pandemic has pushed society towards a dramatic, and often chaotic, adoption of digital health tools. From the implementation of teleconsultations to the embracing of digital solutions in health care, this new paradigm has arrived to stay, following a long-overdue need to embrace digital solutions to improve the care of our patients. Now the question is whether it will be carried out while taking into consideration the unique needs of the rheumatology specialty or, as has happened before, whether we will be forced to use technologies designed by those without an understanding of the particular needs of our complex patients and ourselves as health-care professionals [11]. A relevant finding of this study is that only 37% of the patients are stable users of the digital solution. This fact denotes that multidisciplinary collaboration is crucial in the implementation of digital health in rheumatology; not only are experts in digital health needed, but also professionals in rheumatology and patients, who are the best that may identify patients’ needs. The World Health Organization, in recent guidelines on the use of digital health for strengthening health-care systems [12], strongly highlights the importance of involving health-care professionals early on, in order to address potential implementation issues, and also to include the need for training and addressing digital health literacy issues. Adoption of eHealth can only happen with trust in such systems, and we cannot trust systems that were designed without strong involvement of clinicians and patients.

At this turning point, rheumatologists must seize the challenge to become the leaders of this paradigm shift by becoming involved in the mHealth implementation process. Based on past experience, we do not want to repeat the problems that arose with Electronic Health Records, in which the clerical burden, the lack of interoperability between disparate systems and the non-intuitive interfaces led to significant physician dissatisfaction and a higher risk of professional burnout [13] while failing to facilitate the integrated care that our patients require. In this work, Müskens *et al.* [1] demonstrate that the efficient implementation of digital tools is possible. It shall be a source of inspiration to continue generating evidence on such a necessary and important field. Now is the moment to go the extra mile towards the homogeneous and orchestrated creation of a new paradigm that integrates the digital world and the real world.

## References

[1] Müskens WD, Rongen-van DS, Vogel C et al Telemedicine in the management of rheumatoid arthritis: maintaining disease control with less health-care utilization. Rheumatol Adv Pract 2021;5:rkaa079. 33688619 10.1093/rap/rkaa079 PMC7928564

[2] Smolen JS, Landewé RBM, Bijlsma JWJ et al EULAR recommendations for the management of rheumatoid arthritis with synthetic and biological disease-modifying antirheumatic drugs: 2019 update. Ann Rheum Dis 2020;79:685–99. 31969328 10.1136/annrheumdis-2019-216655

[3] van Tuyl LHD, Sadlonova M, Hewlett S et al The patient perspective on absence of disease activity in rheumatoid arthritis: a survey to identify key domains of patient-perceived remission. Ann Rheum Dis 2017;76:855–61. 27903508 10.1136/annrheumdis-2016-209835

[4] Studenic P, Radner H, Smolen JS, Aletaha D. Discrepancies between patients and physicians in their perceptions of rheumatoid arthritis disease activity. Arthritis Rheum 2012;64:2814–23. 22810704 10.1002/art.34543

[5] Nishiguchi S, Ito H, Yamada M et al Self-assessment of rheumatoid arthritis disease activity using a smartphone application. Development and 3-month feasibility study. Methods Inf Med 2016;55:65–9. 26391694 10.3414/ME14-01-0106

[6] Taylor PC. Adopting PROs in virtual and outpatient management of RA. Nat Rev Rheumatol 2020;16:477–8. 32504074 10.1038/s41584-020-0449-6 PMC7274267

[7] Fernandes A, Van Lenthe FJ, Vallée J, Sueur C, Chaix B. Linking physical and social environments with mental health in old age: a multisensor approach for continuous real-life ecological and emotional assessment. J Epidemiol Community Health 2021;75:477–83. 33148684 10.1136/jech-2020-214274 PMC8053354

[8] Shiffman S, Stone AA, Hufford MR. Ecological momentary assessment. Annu Rev Clin Psychol 2008;4:1–32. 18509902 10.1146/annurev.clinpsy.3.022806.091415

[9] Weldring T, Smith SMS. Patient-Reported Outcomes (PROs) and Patient-Reported Outcome Measures (PROMs). Heal Serv Insights 2013;6:61–8. 10.4137/HSI.S11093 PMC4089835 25114561

[10] Doyle C, Lennox L, Bell D. A systematic review of evidence on the links between patient experience and clinical safety and effectiveness. BMJ Open 2013;3:e001570. 10.1136/bmjopen-2012-001570 PMC3549241 23293244

[11] Menachemi N, Ford EW, Beitsch LM, Brooks RG. Incomplete EHR adoption: late uptake of patient safety and cost control functions. Am J Med Qual 2007;22:319–26. 17804391 10.1177/1062860607304990

[12] World Health Organization. WHO guideline: recommendations on digital interventions for health system strengthening. https://www.who.int/publications/i/item/9789241550505 (date last accessed, 6 June 2019). 31162915

[13] Shanafelt TD, Dyrbye LN, Sinsky C et al Relationship between clerical burden and characteristics of the electronic environment with physician burnout and professional satisfaction. Mayo Clin Proc 2016;91:836–48. 27313121 10.1016/j.mayocp.2016.05.007

